# Recycling Organic Semiconductors: Toward Sustainable Emerging Electronics

**DOI:** 10.1021/acsmaterialsau.5c00128

**Published:** 2025-10-03

**Authors:** Haechan Park, Yerin Kim, Minji Kim, Jihyeon Jang, Jinyeong Park, Sehyun Kim, Myeonghyeon Na, Kyoseung Sim

**Affiliations:** † Department of Chemistry, 131639 Ulsan National Institute of Science and Technology (UNIST), Ulsan 44919, Republic of Korea; ‡ X-Dynamic Research Center, Ulsan National Institute of Science and Technology (UNIST), Ulsan 44919, Republic of Korea

**Keywords:** organic semiconductors, organic electronics, sustainable electronics, recycling technologies, recyclability-by-design

## Abstract

Organic semiconductors (OSCs) have emerged as essential building blocks for next-generation electronics due to their intrinsic mechanical softness, solution processability, and compatibility with flexible, stretchable, and wearable platforms. However, despite their expanding applications, the environmental and economic implications of OSCs throughout their entire lifecycle, including energy- and solvent-intensive synthesis as well as end-of-life disposal, remain insufficiently addressed. Indeed, the environmental footprint of OSCs is intensified not only by their chemically robust backbones, which resist natural degradation and complicate waste management, but also by synthesis processes that generate large volumes of toxic solvent-based waste. In this Perspective, we first highlight the growing importance of OSC recycling, from both environmental and economic standpoints, as it relates to current organic electronics. We then review recent advances in OSC recycling, encompassing both molecular-level strategies based on chemical depolymerization/repolymerization and materials-level approaches involving selective extraction and reuse. Finally, we discuss the key remaining challenges and propose a critical outlook that emphasizes not only the technical and scientific advancement of OSC recycling technologies but also the adoption of a recyclability-by-design approach. Together, these efforts are essential to enable sustainable organic electronic systems.

## Graphical abstract

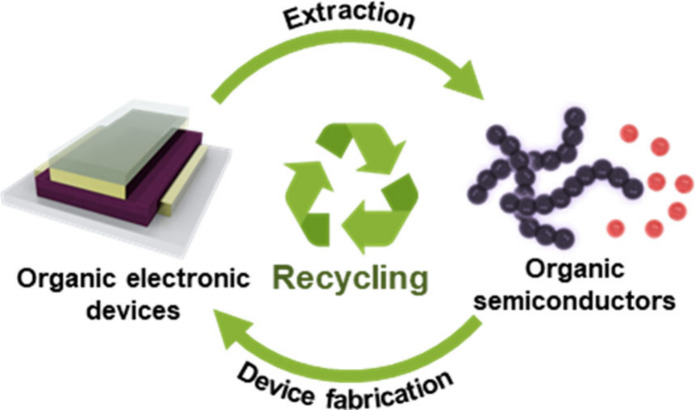

## 1 Introduction

The widespread proliferation of electronic devices has resulted in a sharp increase in electronic waste (e-waste), a significant portion of which is disposed of via landfilling or incineration, leading to resource depletion, environmental contamination, and greenhouse gas emissions.^1^ To date, e-waste concerns have primarily centered on conventional electronics that rely on rigid inorganic materials. However, the advent of organic electronics based on organic semiconductors (OSCs), is redefining both device functionality and end-of-life management in next-generation electronic systems. As key building blocks in organic electronics, OSCs feature solution processability, structural versatility, and mechanical softness, which enable their widespread use in organic thin-film transistors (OTFTs), organic photovoltaics (OPVs), organic light-emitting diodes (OLEDs), and other emerging devices.^2–7^ These materials have already reached commercialization, particularly in OLED displays,^8,9^ and are now being actively expanded into emerging platforms such as stretchable displays, wearable sensors, and implantable bioelectronic systems.^10–13^

This increasing functional diversification of OSCs has intensified concerns about the environmental impact associated with the end-of-life management of organic electronics. Compared to their inorganic counterparts, OSCs inherently suffer from limited operational stability,^14–17^ and are being increasingly integrated into fast-turnover consumer products such as electronic textiles, wearable electronics, and disposable sensors. These emerging applications, often driven by short-lived fashion trends and design-oriented innovation cycles,^11,18,19^ substantially increase the risk of early disposal and large-scale accumulation, thereby accelerating the generation of OSC-related e-waste.^11^ This challenge is further amplified by the maturation of scalable fabrication approaches such as inkjet and roll-to-roll printing. Although these methods effectively reduce production costs, they may also unintentionally shorten product lifespans and increase replacement frequency.^11,20^ Thus, these trends suggest that OSCs are on track to become a significant contributor to the rapidly growing e-waste issues.

Beyond the general concerns related to plastic waste, OSCs present additional environmental challenges due to their unique chemical characteristics. Their conjugated backbones are designed for chemical and thermal stability, rendering them inherently resistant to natural degradation processes. Furthermore, certain OSCs formulations have been reported to exhibit cytotoxicity or to release hazardous intermediates during partial breakdown.^21,22^ Although biodegradable OSCs offer a potential solution, they face limitations including incomplete decomposition and slow degradation.^23–25^ Recycling, by contrast, provides a more scalable and controllable strategy for sustainably managing OSCs.^26^ Unlike biodegradation, recycling enables the recovery and reuse of OSCs before environmental exposure, offering a proactive strategy to mitigate ecological impact while conserving valuable resources. Moreover, recycling directly addresses upstream sustainability concerns related to synthesis of OSCs from both economic and environmental perspectives, which often involves toxic reagents, precious metal catalysts, energy-intensive reactions, and the generation of substantial hazardous chemical waste.^27–29^

Despite the growing promise of OSC recycling, existing systems for recycling electronic materials remain largely unprepared to address it. Current recycling infrastructure and regulations have been primarily developed for the recovery of metals or bulk commodity polymers,^30,31^ offering little consideration for the reclamation of high-value and increasingly prevalent OSCs materials. This systemic gap highlights the urgent need for recycling solutions that are specifically tailored to OSCs. Furthermore, as the electronics industry rapidly transitions toward soft, stretchable, and conformable device platforms, recycling approaches must be adapted to accommodate these emerging formats.

In this perspective, we begin by outlining the urgent need for OSC recycling from environmental and economic standpoints. We then review recent advances in OSC recycling strategies, encompassing both molecular-level depolymerization/repolymerization and materials-level recovery/reuse approaches. Finally, we discuss the remaining scientific and practical barriers and outline strategic considerations for establishing scalable, effective recycling pathways that can support the next-generation sustainable organic electronics.

## 2 Necessity of OSC Recycling

To ensure a sustainable lifecycle for organic electronics, the recycling of OSCs must be regarded as an essential pillar, complementing ongoing efforts in eco-conscious molecular design and green synthesis of OSCs. This section presents the pressing rationale for OSC recycling from two interrelated perspectives including environmental impact and economic feasibility, along with detailed and systemic considerations (Figure 1).

**Figure 1:**
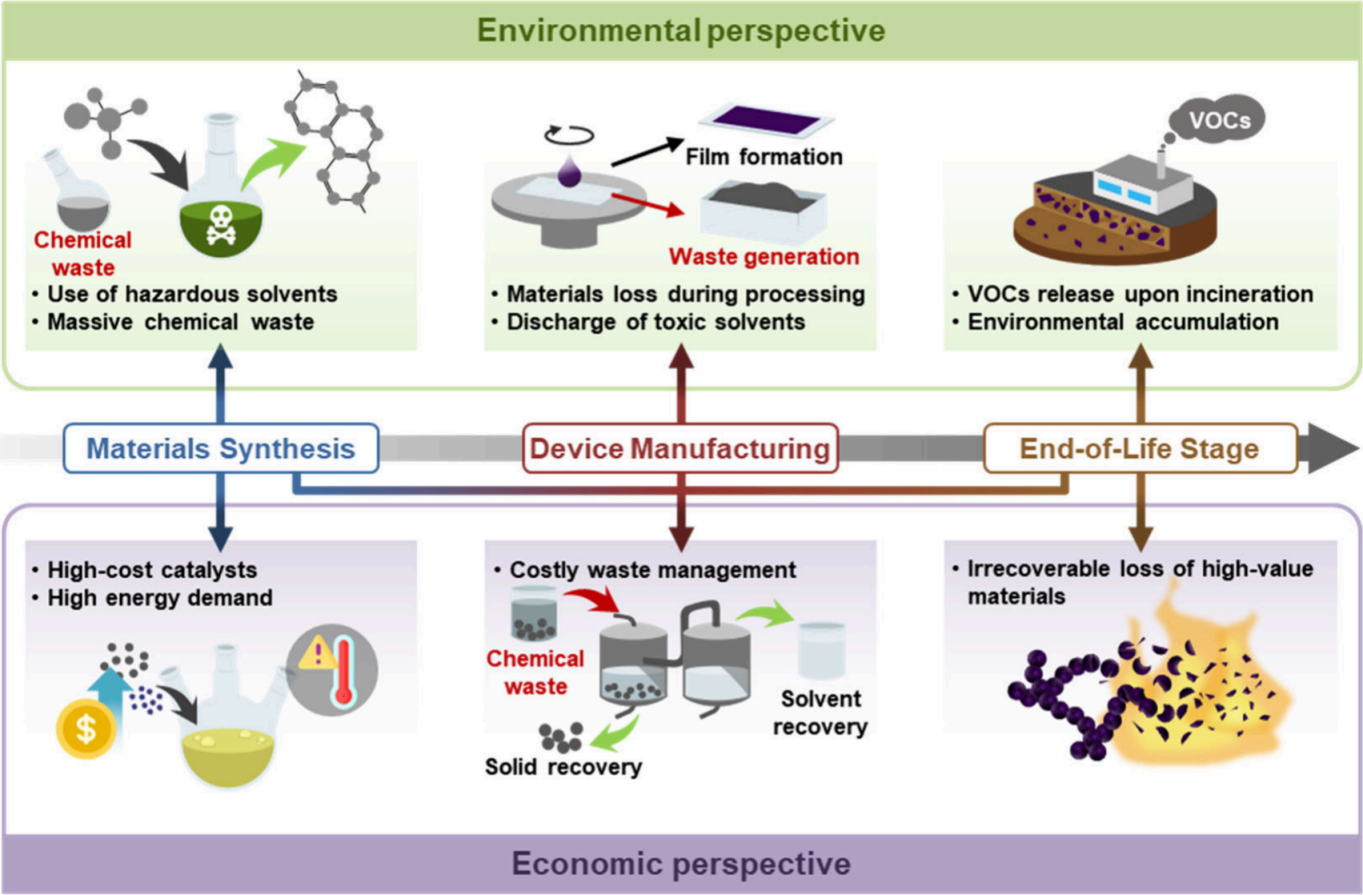
Schematic overview illustrating the necessity of OSC recycling.

### 2.1 Environmental Perspective

The environmental imperative of recycling OSCs arises from their considerable ecological footprint, which spans the entire lifecycle, from material synthesis and device fabrication to end-of-life disposal. Representative polymerization methods for OSCs, including Suzuki polymerization, Stille coupling, and direct arylation polycondensation, commonly rely on large volumes of environmentally hazardous solvents. These processes generate substantial amounts of toxic solvent waste, which involve serious environmental risks due to their volatility, toxicity, and challenges associated with safe disposal.^28,29,32^ For example, the synthesis of poly­(3-hexylthiophene) (P3HT), one of the most widely studied OSCs, has been reported to produce approximately 430 kg of waste per kilogram of product.^33^ In addition, during device fabrication, material utilization is often inefficient. In spin-coating, for instance, only a small fraction of the OSCs solution is deposited onto the substrate, while the majority is discarded as waste, exacerbating environmental concerns. Furthermore, at the end-of-life stage, incineration of OSC-based devices may result in the release of toxic volatile organic compounds (VOCs) similar to conventional plastics, and leads to irreversible loss of the semiconductor materials.^34,35^ This consequently requires iterative synthesis, reinforcing a resource- and waste-intensive production cycle.

Additionally, OSCs present critical end-of-life environmental risks that parallel those associated with conventional plastics, particularly in relation to persistent microplastic accumulation. Their chemically robust conjugated backbones, formed by strong carbon–carbon linkages, render OSCs highly resistant to natural degradation processes, resulting in long-term environmental persistence and potential ecological harm.^21,36^ For instance, OSCs such as dinaphtho­[2,3-b:2′,3′-f]­thieno­[3,2-*b*]­thiophene (DNTT) and pentacene (both consisting of polycyclic aromatic hydrocarbons) have demonstrated intrinsic cytotoxicity, raising concerns regarding their impact on aquatic and terrestrial ecosystems.^22^ While recent efforts have focused on the development of biodegradable OSCs as an alternative,^23–25^ major challenges remain. These include incomplete or slow degradation kinetics, the formation of potentially hazardous intermediate byproducts, and trade-offs in electronic performance associated with enhanced degradability.^37^ Moreover, degradability of OSCs in recent studies has been demonstrated only under controlled conditions, rather than under realistic ecological environments, limiting their practicality.^37–41^ In this context, recycling emerges as a more practical and controllable strategy to mitigate toxic waste accumulation, reduce environmental contamination, and promote resource circularity in the field of organic electronics.

### 2.2 Economic Perspective

Considering the economic burden of synthesizing OSCs, recycling emerges as a cost-effective and sustainable alternative. Unlike biodegradation, recycling offers a unique opportunity to recover and reuse the synthetic building blocks of OSCs, or OSCs themselves, thereby providing significant economic benefits. The synthesis of OSCs generally entails multistep reactions that rely on expensive catalysts, most notably palladium-based complexes, and require labor-intensive purification processes.^29^ Moreover, these processes often generate substantial amounts of waste, which require costly post-treatment and management protocols.^42,43^ These synthetic demands, coupled with the need for precise temperature control to modulate reaction kinetics, result in considerable energy consumption and elevated production costs. The price of certain OSCs has been reported to exceed 450 dollars per gram,^33^ further diminishing the overall economic efficiency. Despite constituting only a minor portion of the total device mass, the OSC layer contributes significantly to manufacturing costs due to its high unit price. For instance, in commercial-scale OPV modules, the active semiconductor material alone can account for more than 35% of total production expenditures.^44^ Therefore, establishing effective recycling pathways for OSCs would not only enable the recovery and reuse of high-value materials but also reduce the frequency of costly synthesis processes, thereby minimizing raw material consumption and lowering overall production costs. Beyond direct economic savings, such strategies offer the potential to alleviate supply chain vulnerabilities stemming from limited material availability and fluctuations in catalyst pricing, particularly those involving precious metals. By enhancing resource security and cost predictability, recycling can strengthen the economic resilience and long-term stability of organic electronics manufacturing. Consequently, the development of economically viable recycling technologies is not only desirable but essential for ensuring the scalable and sustainable growth of the OSCs industry.

## 3 Current Advances in OSC Recycling Strategies

While the field of OSC recycling remains in its early stages, a few pioneering strategies have begun to emerge. These approaches can be broadly classified into two complementary pathways, including (i) molecular-level recycling, which entails the complete depolymerization of polymeric OSCs into their constituent monomers followed by repolymerization, and (ii) materials-level recycling, which involves the selective recovery and direct reuse of OSCs materials from devices with minimal postprocessing. This section highlights representative examples of each strategy, focusing on their chemical principles, processing methodologies, and implications for subsequent device performance.

### 3.1 Molecular-Level OSC Recycling

Molecular-level recycling of OSCs, which involves rapid and complete chemical depolymerization of polymeric OSCs with intentionally controlled manner into their constituent monomers followed by repolymerization, has recently emerged as a promising and sustainable strategy for material circularity.^45^ This approach conceptually parallels the chemical recycling of commodity plastics, where polymers are broken down into monomers and subsequently resynthesized.^46,47^ However, OSCs require substantially higher standards for monomer purity and backbone integrity to preserve electronic functionality upon reuse. To enable selective depolymerization, cleavable linkages could be intentionally embedded within the polymer backbone, allowing for controlled disassembly in response to specific stimuli such as heat or hydrolytic conditions (Figure 2). In principle, this approach allows for multiple recycling cycles without compromising intrinsic electronic properties of the materials, as long as high purity and fidelity is maintained, thereby offering a viable pathway toward closed-loop sustainability in organic electronics.

**Figure 2:**
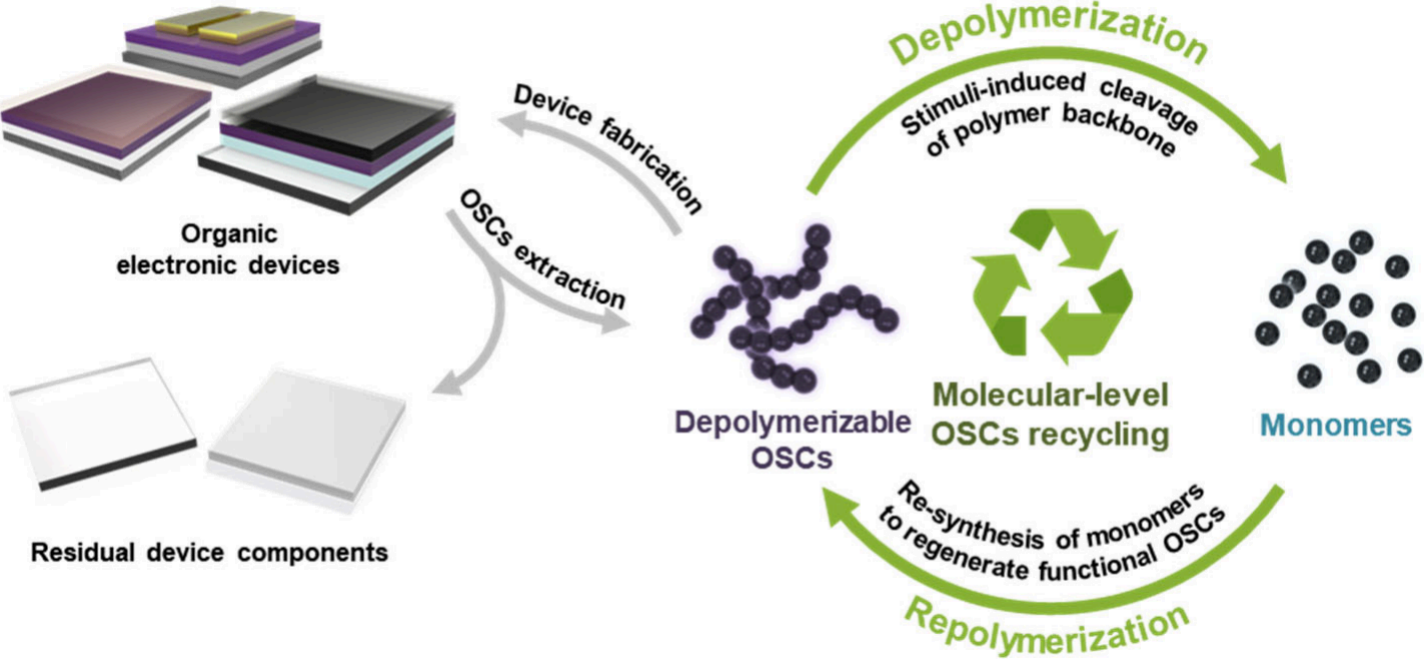
Schematic illustration of the molecular-level recycling of OSCs.

Jin et al. reported one of the earliest demonstrations of molecular-level OSC recycling by developing a depolymerizable conjugated polymer, PY-TIP, which incorporates acid-labile imine bonds within its backbone.^48^ This polymer was employed as an electron acceptor in inverted bulk-heterojunction OPVs. Upon exposure to mild acidic conditions, PY-TIP underwent efficient hydrolytic depolymerization into its monomeric precursor, Y5-TA, which was subsequently repolymerized into PY-TIPO and reused in the fabrication of new OPV devices (Figure 3a). The recycling process began with dissolution of the OSC layer from the device, followed by a series of separation and purification steps including filtration. The recovered monomer exhibited high chemical purity, sufficient for repolymerization into the functional OSCs. This study represents the first successful realization of monomer-level depolymerization and repolymerization of a conjugated polymer directly retrieved from a functional device, establishing a foundational precedent for molecular-level recycling in practical organic electronic systems.

**Figure 3:**
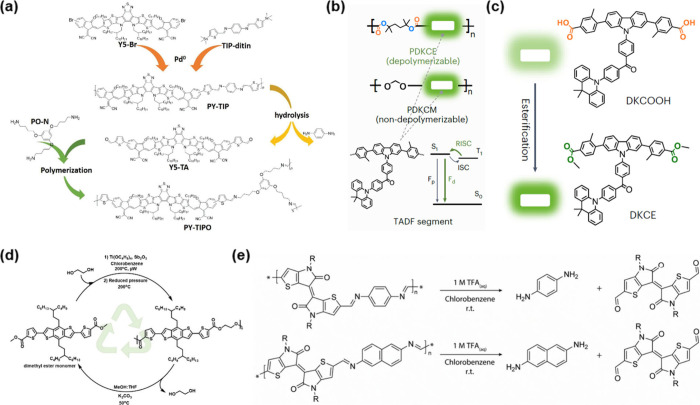
Example of the molecular-level recycling of OSCs. (a) Schematic diagram of preparing the recyclable conjugated polymer, PY-TIP, and the recycling process. Reproduced with permission under a Creative Commons CC-BY 4.0 license from ref 48. Copyright 2023 Jin et al. (b) The chemical structures of the depolymerizable TADF polymer, PDKCE, and its control polymer. (c) Recycling of the thermally depolymerized moiety from PDKCE as DKCE by esterification. Reproduced with permission from ref 49. Copyright 2024 Springer Nature. (d) Closed-loop recycling of PBnDT-TEET. Reproduced with permission from ref 50. Copyright 2024 John Wiley and Sons. (e) Synthetic schemes illustrating the polymer degradation of p­(TII-PD) and p­(TII-2,6ND). Reproduced with permission under a Creative Commons CC-BY 3.0 license from ref 47. Copyright 2024 Nozaki et al.

Liu et al. developed a thermally activated delayed fluorescence (TADF) polymer, PDKCE, specifically designed for OLED applications.^49^ The polymer backbone was engineered to incorporate ester linkages, enabling two distinct depolymerization routes (Figure 3b). Under acid-catalyzed hydrolysis, over 90% of the polymer was converted into oligomeric fragments within 4 days. Alternatively, thermal depolymerization produced a monomeric dicarboxylic acid (DKCOOH) with high structural integrity and sufficient purity for direct reuse, achieving almost 100% yield without further purification. The recovered monomer was subsequently esterified to regenerate DKCE, which can be used in OLED applications (Figure 3c). Additionally, the depolymerized monomer can potentially be repolymerized after isolation. This underscores that the integration of cleavable chemical linkages into the OSC backbone not only enables efficient monomer recovery, but also supports the potential for closed-loop recycling while maintaining the optoelectronic properties essential for high-performance device applications.

Recently, Megret-Bonilla et al. developed ester-functionalized polymeric OSCs by embedding cleavable ester linkages into the polymer backbone to enable molecular-level recycling via methanolysis.^50^ Three copolymers (PBnDT-TEET, PBnDT-TEMT, and PBnDT-TET) were synthesized via Stille coupling between a benzodithiophene (BnDT) core and ester-functionalized comonomers. Among these, PBnDT-TEET exhibited the highest recyclability, yielding a single dimethyl ester–terminated monomer with an isolated yield of 92.3%. This monomer was subsequently repolymerized via transesterification polycondensation to regenerate PBnDT-TEET, enabling closed-loop cycle of OSCs (Figure 3d). Although the recycled polymer yielded limited photovoltaic performance (power conversion efficiency of 0.03%), this work demonstrates the feasibility of designing chemically closed-loop recyclable OSCs through well-considered incorporation of cleavable motifs. It also highlights the intrinsic trade-off between device performance and recycling efficiency.

Nozaki et al. synthesized two imine-linked, thienoisoindigo (TII)-based polymeric OSCs, p­(TII-PD) and p­(TII-2,6ND), designed for recycling through acid-catalyzed depolymerization (Figure 3e).^47^ The polymers were synthesized through polycondensation between a dialdehyde-functionalized TII monomer and aromatic diamines. Upon exposure to acidic conditions, both polymers underwent efficient depolymerization, yielding the original TII monomer with over 90% isolated yield. The recovered monomer was subsequently repolymerized to regenerate the original polymers, enabling successful closed-loop recycling. Importantly, the field-effect transistor fabricated from the recycled polymer exhibited performance comparable to that of its pristine counterpart, confirming the preservation of electronic functionality after recycling. This work underscores the feasibility of molecular-level OSC recycling strategies that simultaneously achieve closed-loop material recovery and retention of device-level performance.

### 3.2 Materials-Level OSC Recycling

Materials-level recycling presents a practical and potentially scalable pathway for the sustainable management of OSCs by enabling the direct recovery of intact OSC layers from disassembled devices. Unlike molecular-level strategies that rely on complete depolymerization and subsequent repolymerization, this approach preserves the original chemical structure and electronic functionality of the OSCs. Among various methodologies, solvent-selective extraction has emerged as a particularly promising technique. In this strategy, orthogonal solvents are employed to selectively dissolve and isolate the OSC layer without disrupting adjacent device components, such as electrodes or dielectric layers (Figure 4). This solvent orthogonality facilitates layer-specific retrieving while minimizing cross-contamination, thereby enabling high-purity material reuse in subsequent fabrication cycles.

**Figure 4:**
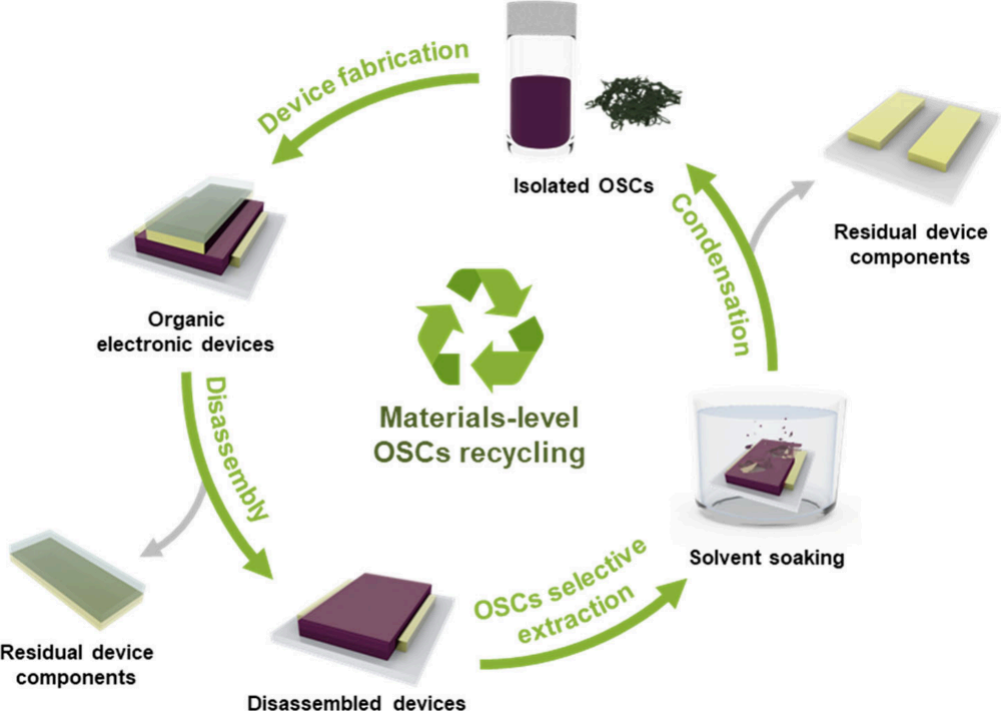
Schematic illustration of the materials-level recycling of OSCs.

Our recent study demonstrated a world first materials-level OSC recycling strategy through the development of recyclable organic flexible (ROF) electronics, in which P3HT was selectively extracted and directly reused as a polymeric OSC.^26^ Following device disassembly via physical removal of the ion gel dielectric layer, P3HT was selectively dissolved using an orthogonal solvent that did not affect the adjacent organic conductor, poly­(3,4-ethylenedioxythiophene):poly­(styrenesulfonate) (PEDOT:PSS) (Figure 5a). The extracted solution was concentrated by rotary evaporation, during which both the polymer and the solvent were recovered for reuse. The recycled P3HT formed films with morphologies comparable to those of pristine P3HT (Figure 5b). Importantly, the recovered material was directly reprocessed into new organic electrochemical transistors (OECTs), which exhibited electrical performance equivalent to that of devices fabricated using pristine P3HT (Figure 5c). This result confirms the practical viability of selective solvent extraction as a materials-level recycling strategy for flexible organic electronics.

**Figure 5:**
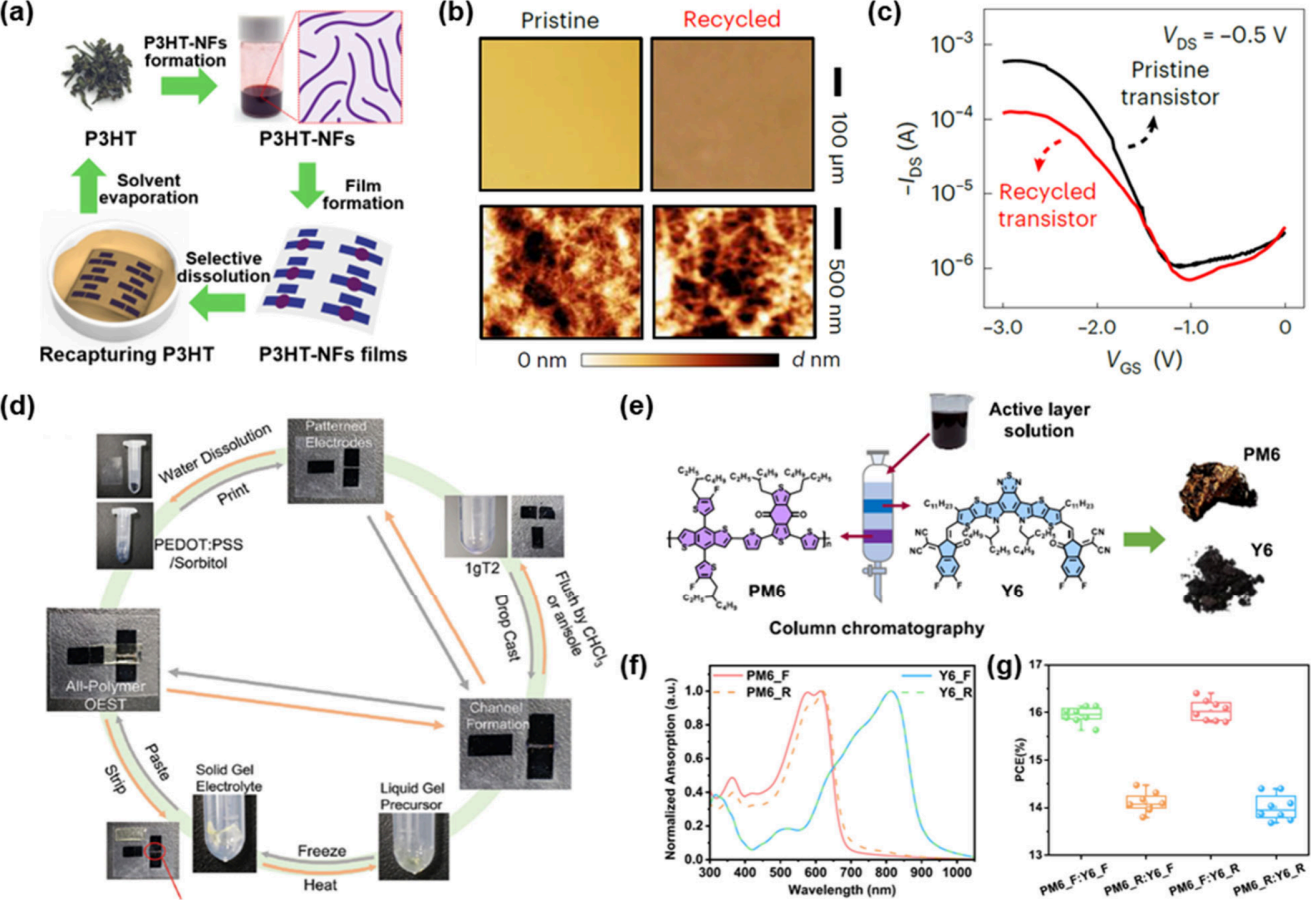
Example of the materials-level recycling of OSCs. (a) Schematic of the solvent-selective extraction process of P3HT. (b) Optical microscopic images (top) and AFM images in height mode (bottom) of pristine and recycled P3HT. (c) Transfer curves of pristine and recycled OECTs, in which pristine and recycled P3HT were employed as the active layers of each device, respectively. Reproduced with permission from ref 26. Copyright 2023 Springer Nature. (d) Assembly and recycling processes of the OESTs enabling solvent-selective extraction of P3gCPDT-1gT2. Reproduced with permission from ref 51. Copyright 2024 John Wiley and Sons. (e) Schematic purification process of selectively extracted active-layer materials from the devices into PM6 and Y6. (f) UV–vis absorption spectra of fresh (denoted as “_F”) and recycled (denoted as “_R”) PM6 and Y6. (g) Summarized PCEs of OPVs constructed with fresh and recycled PM6 and Y6. Reproduced with permission from ref 44. Copyright 2024 Elsevier.

Building upon this approach, He et al. applied selective extraction to recover P3gCPDT-1gT2 from fully recyclable organic electrochemical synaptic transistors (OESTs).^51^ In this study, the gelatin-based organohydrogel electrolyte was first removed by mechanical peeling, after which the underlying OSC layer was selectively dissolved using an orthogonal solvent (Figure 5d). This process enabled efficient separation of the semiconducting material without damaging the electrode structure. Although electrical characterization of devices incorporating the recycled OSC was not reported, this work highlights the compatibility of selective extraction strategies with neuromorphic device architectures and supports their broader applicability across functional platforms.

More recently, Sun et al. developed a scalable and economically viable recycling protocol for OPVs utilizing PM6:Y6 active layers.^44^ After disassembling the multilayer device stack, the active layer was selectively dissolved in toluene, followed by purification steps including column chromatography to remove residual impurities (Figure 5e). The recovered donor and acceptor materials, PM6 and Y6, were obtained with yields of 84.2% and 73.5%, respectively, and their chemical structures were preserved, as confirmed by ultraviolet–visible (UV–vis) absorption spectra (Figure 5f). The recycled materials were then reprocessed into new OPV devices that exhibited power conversion efficiencies comparable to those of devices fabricated with pristine materials (Figure 5g). Furthermore, an economic analysis estimated potential cost savings of $14.24 per m^2^, underscoring the industrial relevance and scalability of this materials-level recycling approach.

## 4 Key Challenges in OSC Recycling

Despite increasing interest and recent progress in the development of recycling strategies for OSCs, a range of technical, structural, and systemic challenges continue to impede their widespread adoption. This section examines current recycling methodologies within the broader framework of molecular-level and materials-level approaches, identifying their respective limitations. In particular, we discuss the fundamental barriers that must be addressed to enable sustainable and scalable OSC recycling, with emphasis on their integration into future electronic device platforms.

### 4.1 Limitations of Current OSC Recycling Strategies

Molecular-level recycling of OSCs, which involves the complete depolymerization of polymeric semiconductors into monomers followed by repolymerization, holds strong potential for achieving closed-loop sustainability. However, current approaches rely heavily on multistep synthetic protocols that involve toxic reagents, precious-metal catalysts, and solvent-intensive purification procedures. These requirements can generate substantial chemical waste, potentially offsetting the environmental benefits of recycling unless greener alternatives, such as solvent-free polymerizations or catalyst-free coupling reactions, are adopted. In addition, achieving high-purity monomer recovery remains inherently challenging. Even in successful depolymerization cases, such as that reported by Megret-Bonilla et al.,^50^ chromatographic purification is typically required before the repolymerization step to restore performance of the material to acceptable levels. Furthermore, the long-term recyclability of these systems is largely unproven. That is, most demonstrations are limited to single-cycle recycling studies, leaving key concerns unresolved, including impurity buildup, unintended polymer chain degradation during depolymerization, and limited process reproducibility. A particularly critical challenge lies in regenerating the original polymer structure from depolymerized monomers over multiple cycles. In many cases, the depolymerization process yields structurally altered derivatives rather than the exact monomer units, making it difficult to reconstruct the initial polymer architecture. This limitation significantly constrains both the fidelity and scalability of molecular-level recycling and presents a major barrier to its broader implementation.

Materials-level OSC recycling, which typically relies on selective layer extraction using orthogonal solvents, provides a more scalable and device-compatible alternative. However, in practical applications, unintentional codissolution of adjacent layers frequently occurs, leading to contamination and a corresponding reduction in the purity of the recovered semiconducting material. This, in turn, necessitates additional purification steps that increase both environmental impact and operational complexity.

Both molecular- and materials-level strategies require the complete disassembly of multilayer device stacks, a step often overlooked in laboratory-scale demonstrations. In reality, however, this disassembly process is often highly complex, involving multiple steps such as mechanical delamination, selective chemical etching, and component separation.^52^ These procedures can inadvertently damage critical components such as substrates, electrodes, or encapsulation layers, thereby complicating reuse and undermining the recyclability of the full system. For future applications involving soft, stretchable, or implantable OSC-based devices, the development of simplified and nondestructive disassembly methods will be essential.

### 4.2 Inherent Structural and Architectural Challenges

Organic electronic devices are typically constructed as multilayered thin-film stacks comprising electrodes, OSCs, dielectrics, substrates, and encapsulation layers. These layers are typically bound by a combination of physical adhesion and chemical interactions, such as interfacial reactions, cross-linking chemistry, and blended interlayers, that hinder layer separation during recycling.^53^ Additionally, in many cases, complex architectures involving binary or ternary blend systems are employed to enhance charge transport, improve interfacial adhesion, or introduce additional functionalities.^54,55^ While such architectures enhance initial device performance and support multifunctionality, they significantly impede the selective separation or depolymerization of OSCs during end-of-life recovery.

These challenges are further amplified in soft electronic platforms, which represent a rapidly growing domain for OSCs. In applications such as electronic skin (e-skin), soft robotics, and implantable bioelectronic systems, OSCs are frequently embedded within elastomeric matrices to provide mechanical softness and conformal integration with soft or dynamic surfaces.^13,56^ While this strategy is essential for achieving robust mechanical and functional coupling with biological tissues or moving substrates, it also introduces complex composite morphologies characterized by phase intermixing and interpenetrating networks. These complex morphologies are difficult to recycle using existing techniques such as solvent extraction or thermal separation, because the chemically and physically entangled components are hard to isolate.^57^

### 4.3 Systemic and Economic Barriers

Beyond technical challenges, systemic and economic constraints represent critical obstacles to the industrial realization of OSC recycling. Currently, existing recycling infrastructures for e-waste are optimized for bulk plastics and metals, relying heavily on incineration or pyrolysis that inevitably degrade or destroy OSCs, rendering their recovery unfeasible. Unlike metals or commodity plastics, OSC-based devices lack dedicated infrastructure for end-of-life handling, component-level disassembly, and targeted material recovery. As a result, most OSC-related devices are discarded as undifferentiated e-waste, with no classification or processing protocols specific to OSCs. This situation reflects not only the absence of regulatory frameworks such as mandatory guidelines and standardized processing protocols, but also broader lack of momentum within the industry to address the issue.

Furthermore, although laboratory-scale demonstrations have shown promise, the economic feasibility of OSC recycling remains largely unproven. High raw material costs, purification inefficiencies, labor-intensive disassembly procedures, and fragmented supply chains collectively hinder commercial viability. These challenges are further amplified in soft or biointegrated electronics. In such systems, complex device architectures and delicate mechanical interfaces hinder material recovery and significantly raise both technical difficulty and operational cost.

## 5 Conclusion and Outlook

As organic electronics have transitioned from early stage technologies to mainstream applications, most notably demonstrated by the commercialization of OLED displays, their application scope continues to broaden into next-generation soft systems, including wearable, skin-conformal, and biointegrated platforms. Within this rapidly evolving field, the recycling of OSCs has shifted from a peripheral sustainability consideration to a central technological priority. As innovation becomes increasingly tied to environmental responsibility, OSC recycling must extend beyond simple material recovery toward a more integrated and forward-looking design principle. In particular, recycling should be redefined as a holistic strategy that not only restores semiconducting functionality but also remains compatible with diverse device requirements, including, for instance, the mechanical demands specific to soft electronics.

While both molecular- and materials-level strategies have demonstrated feasibility, each continues to face inherent limitations that impede broader applicability. Continued development of these individual approaches remains critical, not only to resolve their respective challenges, but also to enhance their integration with evolving device architectures and scalable fabrication processes.

To address the limitations of current approaches and ensure long-term sustainability, a paradigm shift toward recyclability-by-design is essential (Figure 6). Future efforts should prioritize the codevelopment of OSC materials and device architectures to enable selective disassembly, efficient material recovery, and seamless reintegration without compromising electronic performance or mechanical integrity. A promising approach involves achieving orthogonal solubility across stacked layers, achieved either through molecular engineering to finely tune solvent affinity, or by incorporating reversible cross-linking to impart temporary solvent resistance.^58,59^ These strategies minimize codissolution and interlayer contamination during device processing and recycling, while fully preserving material recyclability. Additionally, another practical challenge lies in the recovery of OSCs from fully encapsulated devices, where the presence of encapsulation layers significantly complicates disassembly. A promising approach involves the use of photoreversible polymer systems or light-degradable adhesives,^60,61^ which enable layer-selective access under mild light exposure, thereby facilitating OSC recycling without compromising device integrity. These strategies are particularly critical for emerging soft electronic systems, where semiconductors are embedded in deformable matrices such as elastomers or hydrogels. These systems require the retention of not only electrical functionality but also mechanical properties after recycling, a dual restoration that remains a key unmet challenge, especially in wearable or implantable devices demanding long-term reliability, biocompatibility, and mechanical durability. To meet these demands, potential solutions include the integration of reversible cross-linkers, cleavable adhesives, or selectively soluble matrices to facilitate scalable disassembly and recovery.^62–64^ Ultimately, the development of OSCs that preserve their functional performance across multiple lifecycles is a foundational requirement for sustainable soft electronics.

**Figure 6:**
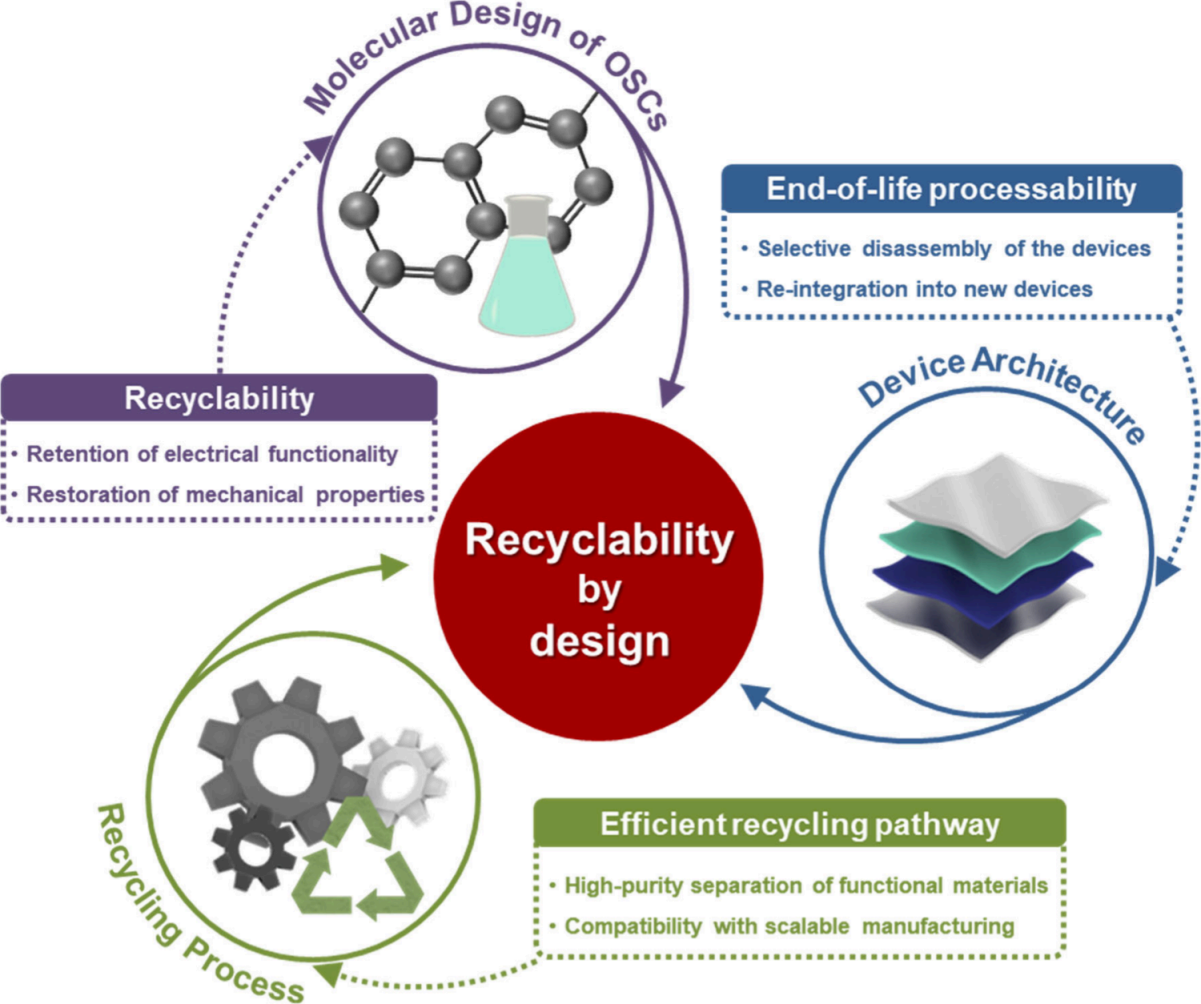
Requirements for recyclability-by-design in future organic electronics through coengineering of materials, devices, and recycling strategies.

Addressing this challenge will require a coordinated advancement in three key areas. First, molecular design of OSCs must simultaneously target recyclability, softness, and stretchability. Chemistries incorporating cleavable yet mechanically resilient linkages, such as dynamic covalent bonds or thermally reversible motifs, may provide viable solutions. Second, device architectures must be adapted to accommodate end-of-life processing through features such as modular construction, solvent-responsive interfaces, or peelable encapsulation schemes. Third, any recycling process must enable high-purity separation of functional materials, retain device-level performance postrecovery, and remain compatible with scalable manufacturing protocols such as printing or roll-to-roll processing.

In addition to advances in molecular and architectural design, systemic and policy-level support will be critical to translating OSC recycling into industrial practice. Coordinated regulatory frameworks should promote extended producer responsibility (EPR), incentivize recyclable-by-design strategies, and enforce traceability standards for materials throughout the product lifecycle. Governments and industry consortia should define classification standards for OSC-based devices and develop dedicated infrastructure for their collection and separation, tailored specifically to organic electronics. These measures will facilitate high-purity material recovery while reducing the environmental footprint of current disposal practices. In parallel, public and private investment should be directed toward scaling up lab-proven recycling techniques. Early collaboration among researchers, device manufacturers, and waste management stakeholders is essential to bridge the gap between academic innovation and industrial application.

In conclusion, the future of OSC recycling depends on the convergence of materials chemistry, device engineering, manufacturing science, and policy development. Realizing fully recyclable, soft, and functional organic electronics is no longer a distant aspiration. Rather, it is a foundational requirement for building a truly sustainable future for electronic materials and devices.
